# Supplementary material for: Neonatal antibiotic exposure impairs child growth during the first six years of life by perturbing intestinal microbial colonization
Source: Nat Commun. 2021 Jan 26;12:443. doi: 10.1038/s41467-020-20495-4 (PMC7838415; doi:10.1038/s41467-020-20495-4)
Supplement: Supplementary file 1 — Supplementary Information [file 41467_2020_20495_MOESM1_ESM.docx]

Supplementary Materials

**Neonatal antibiotic exposure impairs child growth during the first six years of life by perturbing intestinal microbial colonization**

**Authors:** Atara Uzan-Yulzari, Olli Turta, Anna Belogolovski, Oren Ziv, Christina Kunz, Sarah Perschbacher, Hadar Neuman, Edoardo Pasolli, Aia Oz, Hila Ben-Amram, Himanshu Kumar, Helena Ollila, Anne Kaljonen, Erika Isolauri, Seppo Salminen, Hanna Lagström, Nicola Segata, Itai Sharon, Yoram Louzoun, Regina Ensenauer, Samuli Rautava, Omry Koren

Correspondence to: samuli.rautava@hus.fi, omry.koren@biu.ac.il

**Supplementary Results**

Prevalence of Bifidobacterium species and strains in the metagenomics samples

Community composition varied across the different metagenomics samples with members of four different phyla detected: Firmicutes, Bacteroidetes, Proteobacteria and Actinobacteria. Community composition varied significantly among the different samples with a total of at least 35 genera (Supplementary Figure 6) and 88 bacterial species detected across all samples (Supplementary Tables 8 and 10). As expected, the average number of species whose relative abundance is 0.1% or higher in the community is higher in the six-month samples compared to the one-month samples for both the antibiotics (20 vs 13 species/sample) and the control (15.8 vs. 10.4 species/sample) groups.

Multiple strains of the same species were identified in two samples, 126_CTR_6M, 166_ABX_6M and 179_CTR_6M, in all cases for *B. longum*. Comparing one- and six-month samples of the same babies revealed that the same strains were present in both samples in all four cases in which the same Bifidobacterium species was identified. For baby 126, the only *B. longum* strain that was detected in the one-month sample is the major strain in the six-month sample with an additional minor strain detected. For baby 128, the same *B. breve*, *B. bifidum* and *B. pseudocatenulatum* strains are present in both samples. This shows that initial colonizers of the infant gut also remain part of the population in the long term.

**A**


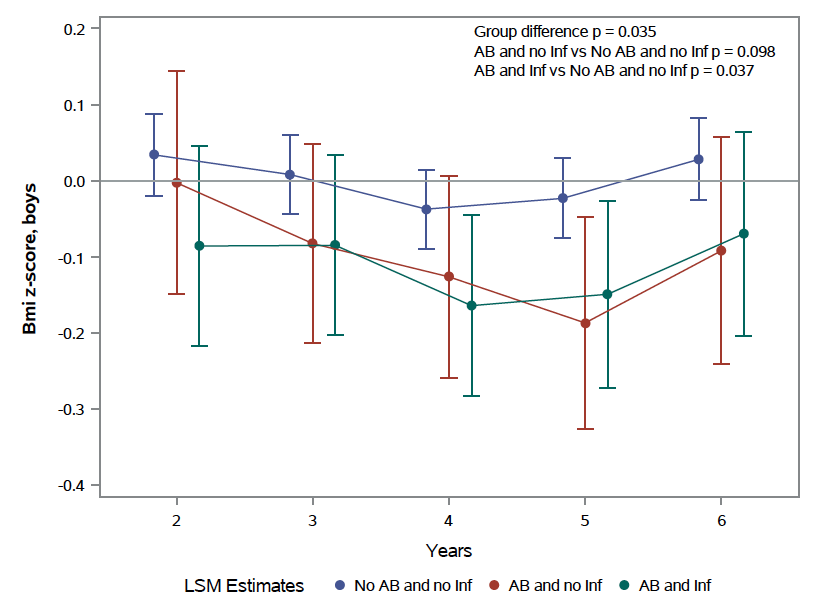


**B**

**
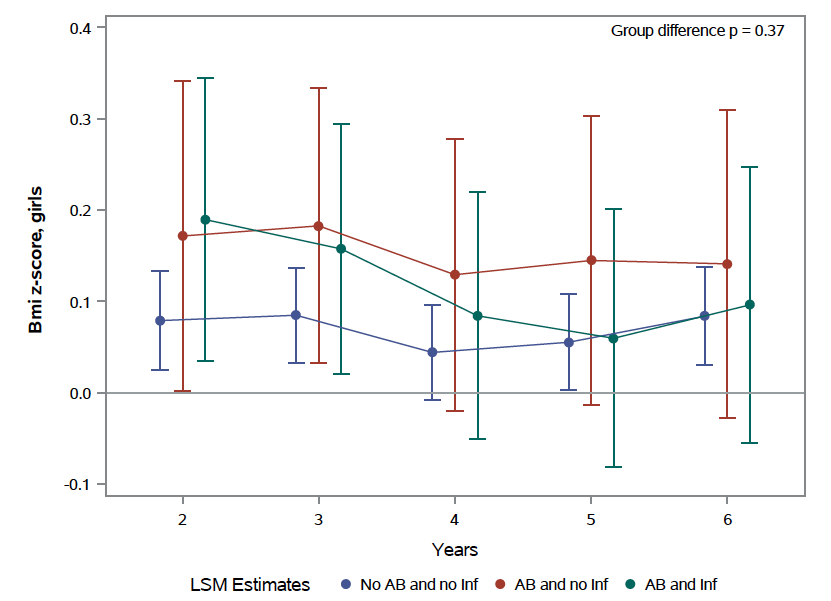
**

**Supplementary Figure** **1. Body mass index (BMI) Z-scores in children exposed to brief empirical antibiotic therapy** (AB and no Inf, N=513) **and children who had received antibiotics for confirmed or clinical bacterial infection** (AB and Inf, N=638) **as compared to children not exposed to antibiotics** (No AB and no Inf, N=11,271)**.** The BMI Z-scores between the ages of 2 and 6 years are presented for boys (**A**) and for girls (**B**). The x axis represents the age in years, the y axis represents the model-based Least Squares Mean (LSM) estimates. The whiskers represent 95% confidence intervals. The data were analyzed using a hierarchical linear mixed model for repeated measurements. Neonatal antibiotic exposure, gestational age, birth weight Z-score, mode of delivery, time, maternal prepregnancy BMI and intrapartum antibiotic treatment were included in the model as explanatory variables.

**A B**


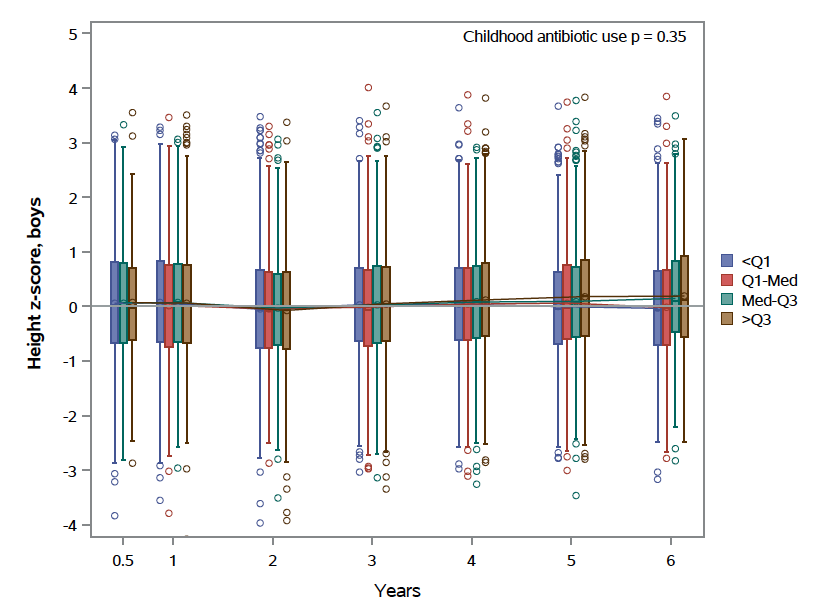

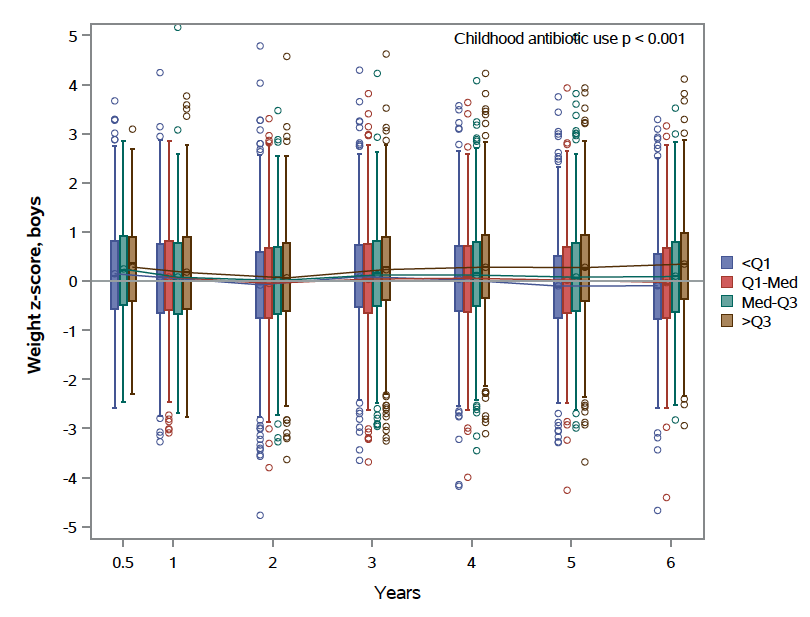


**C D**


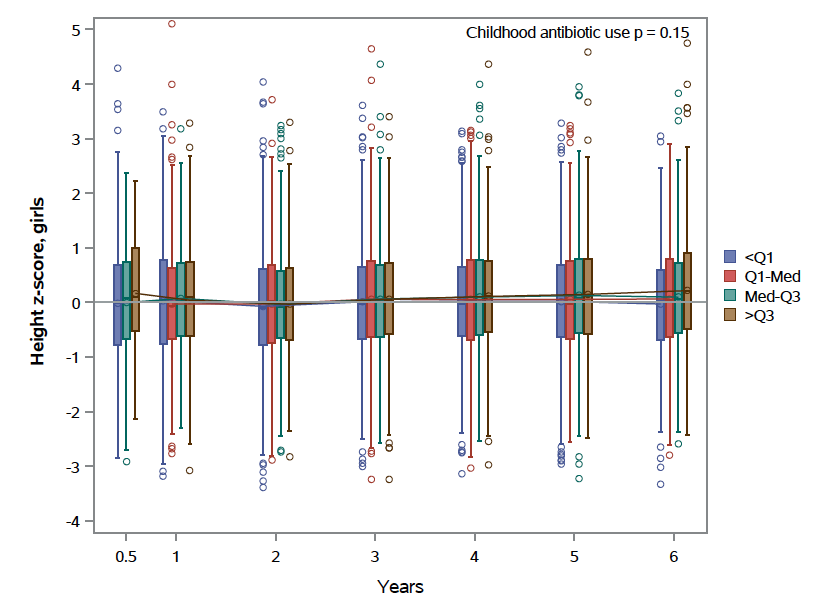

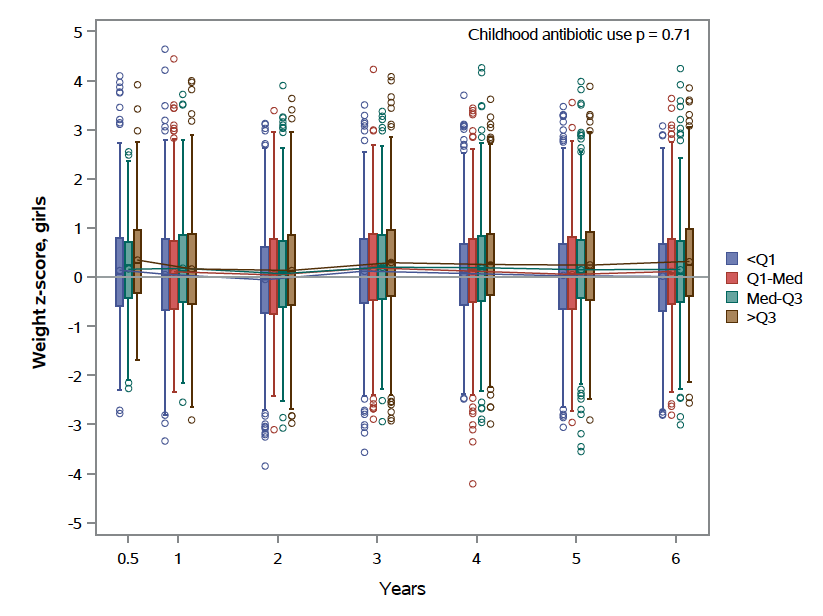


**Supplementary Figure** **2. The association between childhood antibiotic use and weight and height Z-scores during the first six years of life in boys and girls.** The subjects have been categorized by quartiles (Q1, median and Q3) based on the cumulative number of antibiotic purchases at each point in time. The number of antibiotic purchases was associated with significantly higher weight Z-scores during the first six years of life in boys (**A**, p<0.001) but not in girls (**C**, p=0.71) in a hierarchical linear mixed model for repeated measurements adjusted for gestational age, birth weight Z-score, mode of delivery, maternal prepregnancy BMI and neonatal antibiotic exposure. No association between the number of antibiotic purchases and height Z-scores was detected in either boys (**B**, p=0.35) or girls (**D**, p=0.15). The boxes represent interquartile range (IQR) and the whiskers represent 1.5 times IQR. The circles represent outliers.


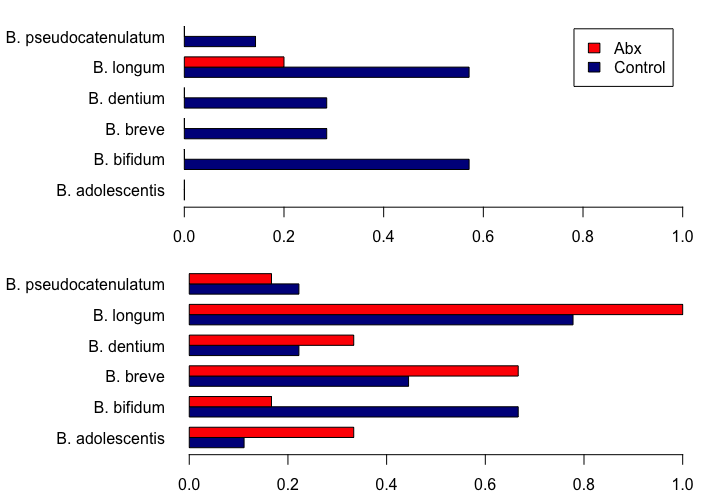


**Supplementary Figure** **3**: Presence of *Bifidobacterium* species in the one-month (top) and the six-month (bottom) samples. Only species with relative abundance ≥ 0.1% were considered. One-month samples: 7 control, 5 antibiotics. Six-months samples: 9 control, 6 antibiotics.


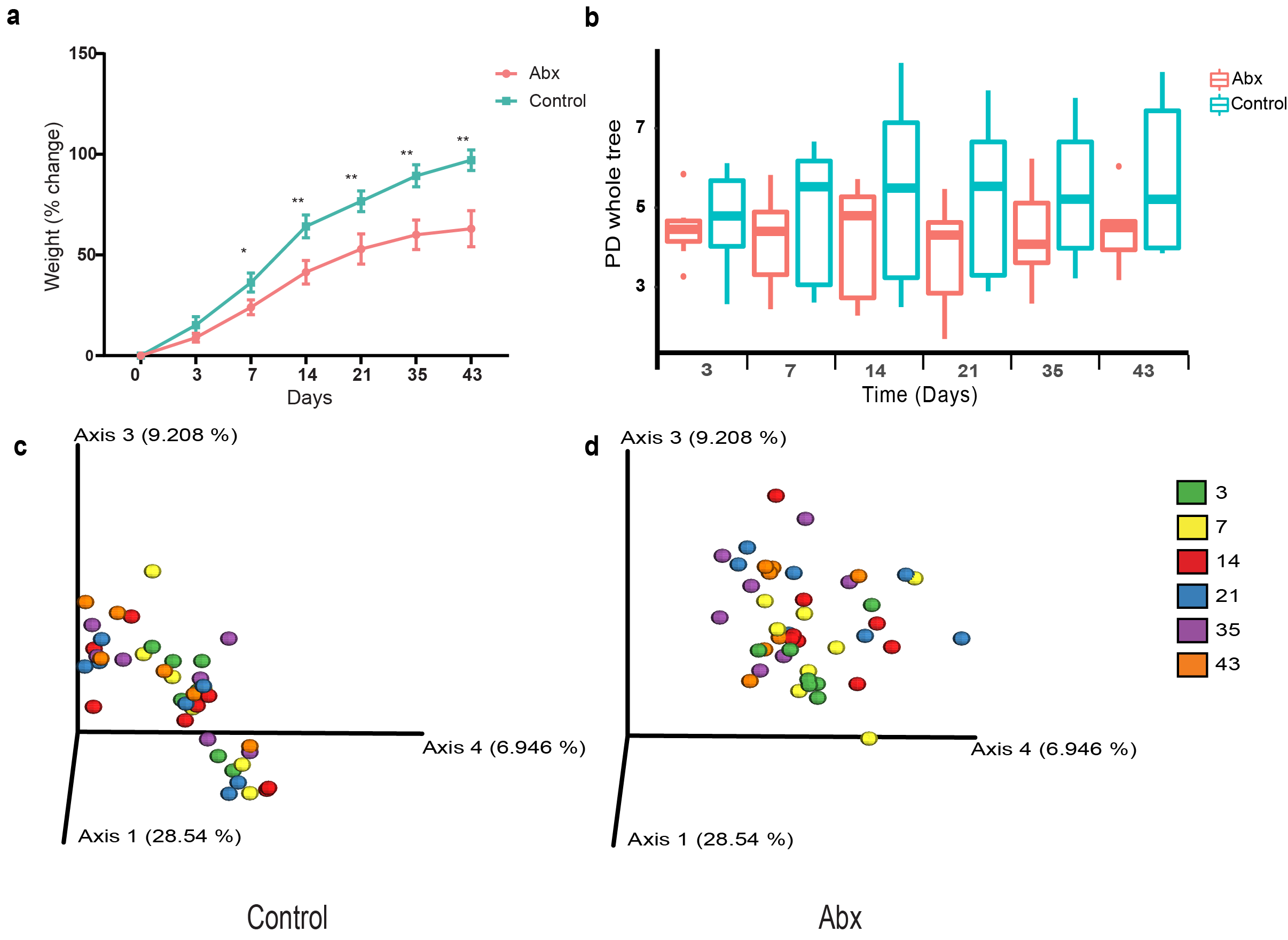


**Supplementary Figure** **4.** **FMT from antibiotic-treated infants, 24 months after exposure, to GF mice induces changes in weight gain and bacterial composition. (A)** Mice receiving fecal transplantation from antibiotic treated infants (pink) gained significantly less weight (expressed as mean +/- SEM) compared to control animals (blue) starting from day 7. **(B)** No significant changes were seen in alpha diversity comparison based on phylogenetic diversity. **(C)** PCoA based on Unweighted UniFrac distance matrix in control, and **(D)** Abx mouse groups at six time points, 3 (green), 7 (yellow), 14 (red), 21 (blue), 35 (purple) and 43 (orange) days from FMT. (For (A) control and abx group, n = 7-8. for (B-D), control group, day 3,7 and 14, n = 8; day 21 and 35, n = 7; day 43, n=6. Abx group, day 3, 21,35 and 43, n=7; day 7 and 14, n = 8). Samples from 3 antibiotic-exposed infants and 7 non-exposed infants were used for fecal transplantation. (**p* < 0.05, ***p* < 0.01)**.**


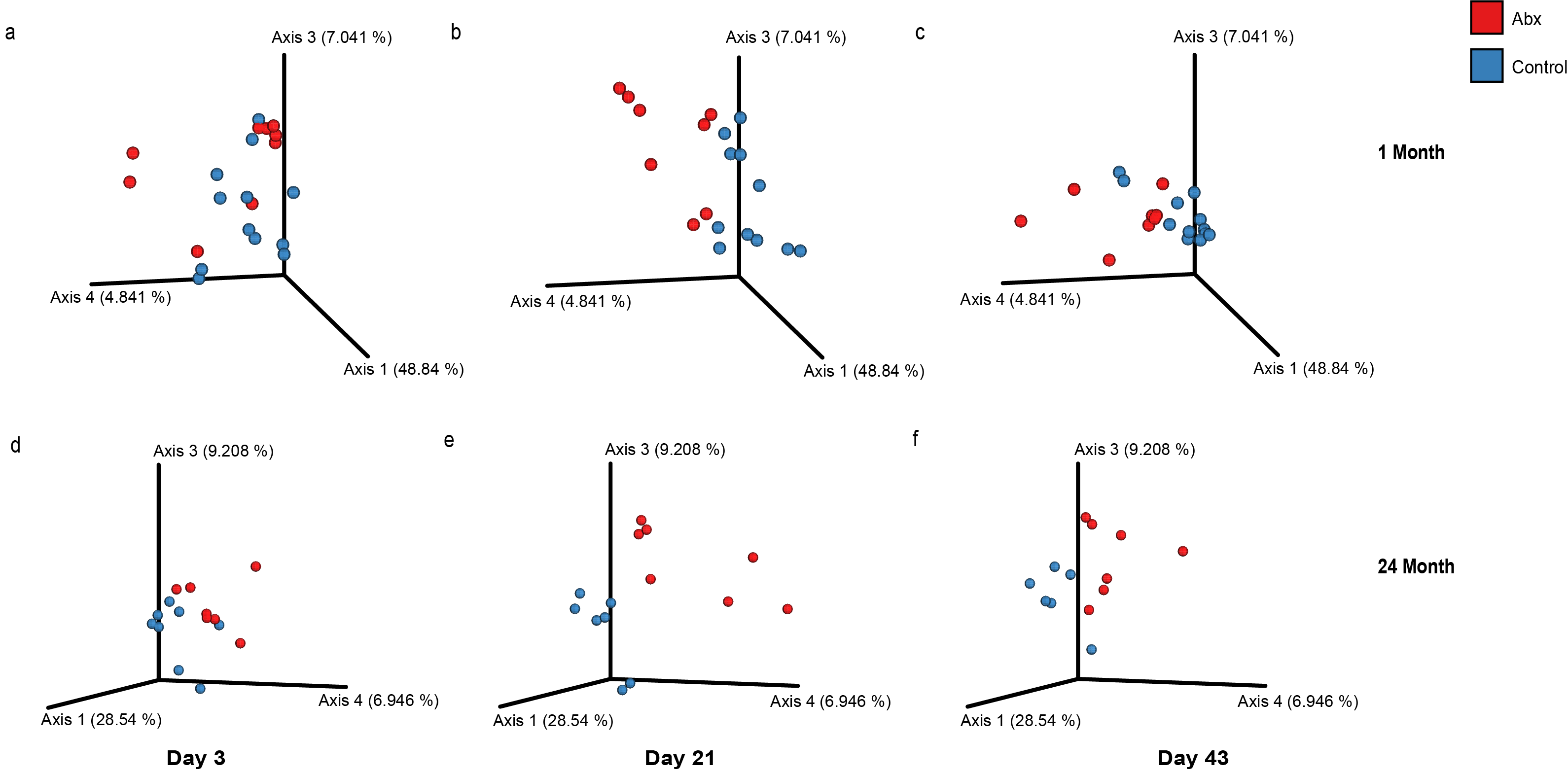


**Supplementary Figure 5.** **FMT from infants to GF mice induces significant changes in bacterial populations.** Phylogenetic beta diversity (unweighted UniFrac) of control (blue) and Abx (red) mice groups over time, 3 (A, control, n=12; abx, n=12 P=0.008, D, control, n=8; abx, n=7, P=0.024), 21 (B, control, n=12; abx, n=8, P=0.006, E, control and abx n=7, P=0.02 ), and 43 (C, control, n=12; abx, n=8 P=0.001, F, control, n=7; abx, n=6, P=0.09) days from fecal transplantation


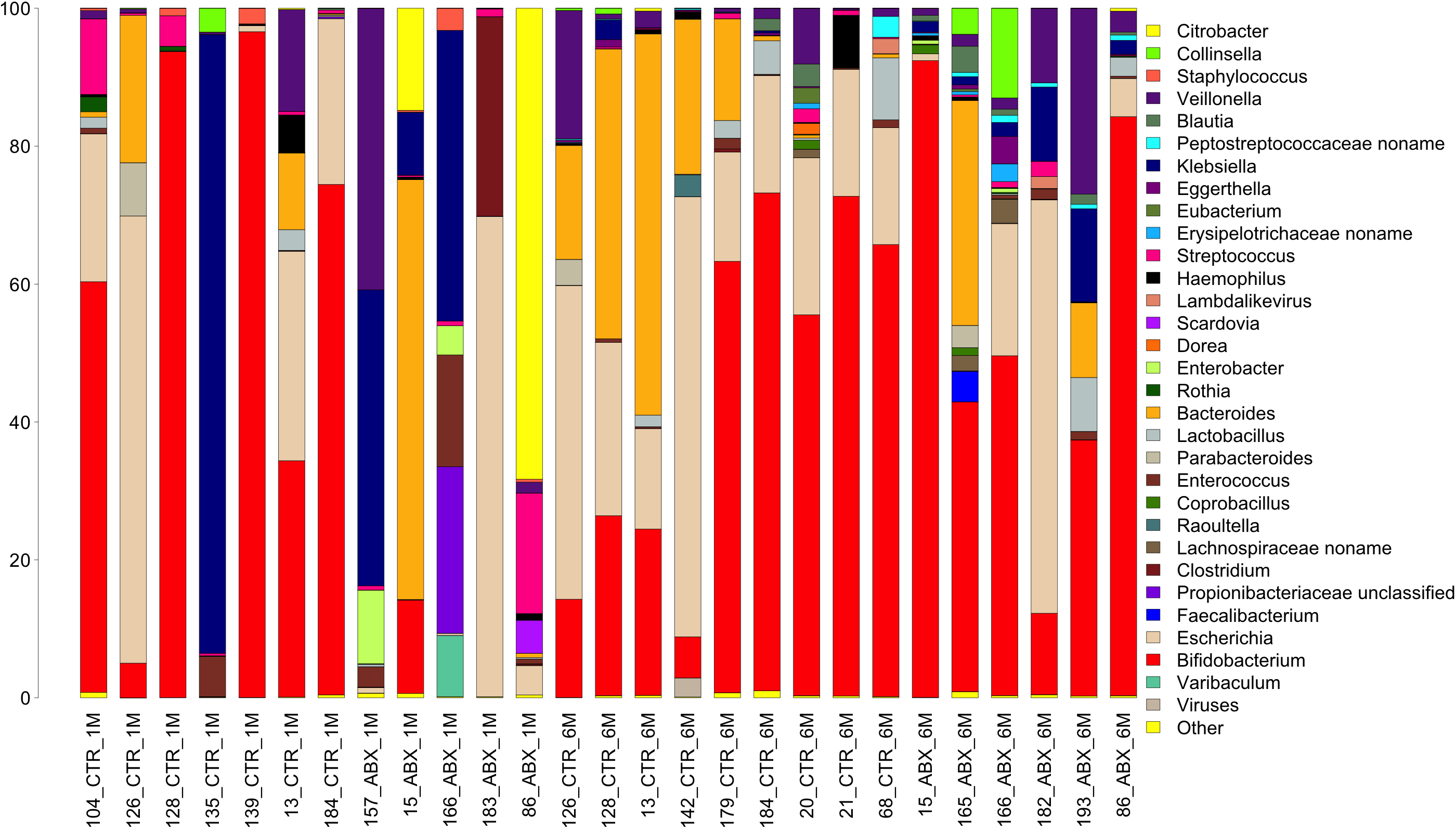


**Supplementary Figure** **6**. **Genus abundance across the different metagenomics samples.** Only genera with relative abundance ≥ 1% in at least one sample are shown. Genus abundance was evaluated using metaphlan2.

**
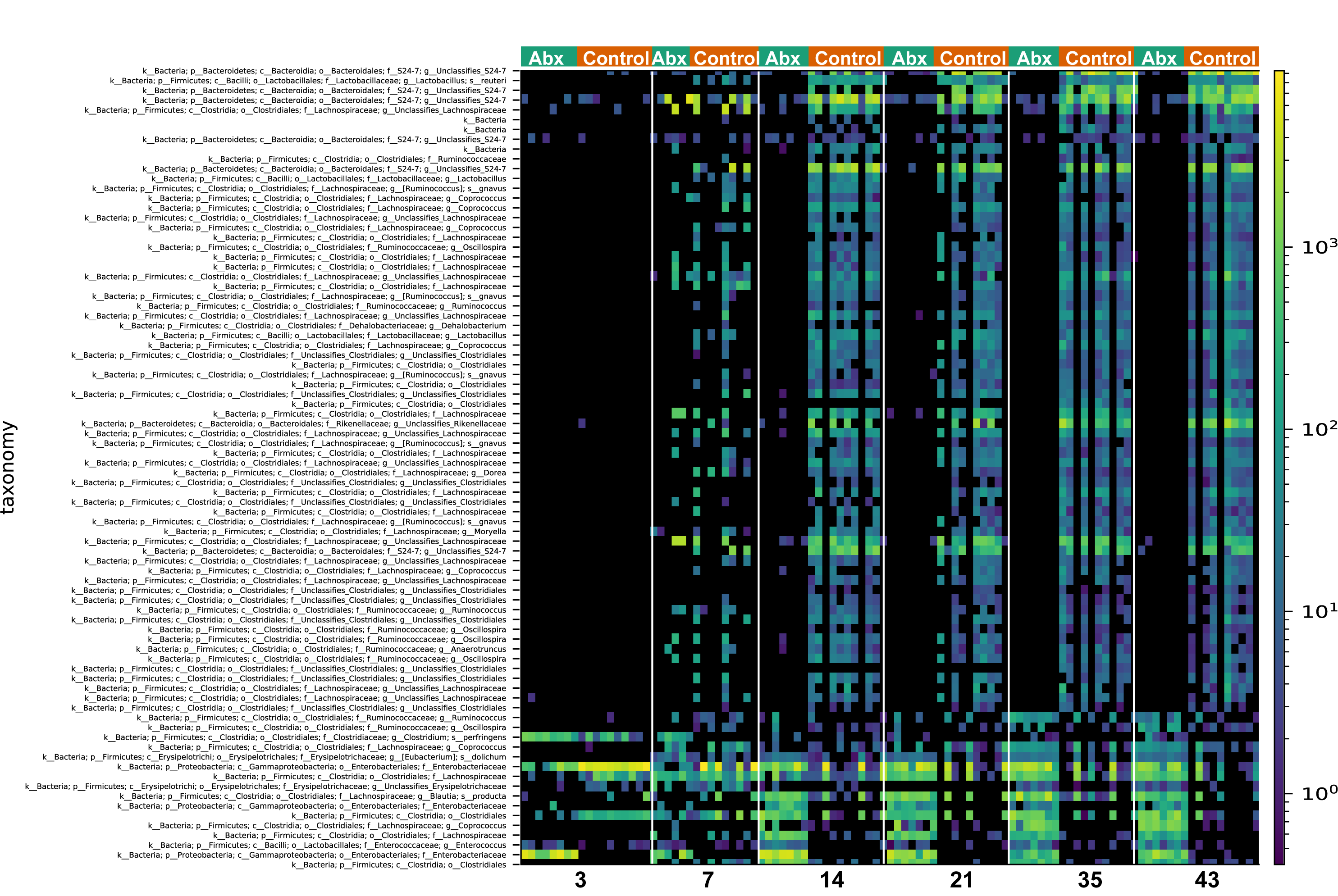
**

**Supplementary Figure** **7 -** A color scale heat map showing significant changes in bacterial composition between control and Abx mice groups on day 43, using Calour (*q* <0.05)

| **Supplementary** **Table 1.** Clinical characteristics of the boys and girls in the Southwest Finland Birth Cohort. | | | |
| --- | --- | --- | --- |
|  | **Boys (N=6307)** | **Girls (N=6115)** | ***p*** |
| Gestational age (weeks) | 40.0 (1.2) | 40.1 (1.2) | 0.0022 |
| Birth weight (grams) | 3636 (469) | 3514 (450) | <0.0001 |
| Birth weight Z-score | 0.03 (1.05) | 0.004 (1.06) | 0.15 |
| Maternal prepregnancy BMI (kg/m^2^) | 24.4 (4.8) | 24.4 (4.7) | 0.70 |
| Vaginal delivery | 87.2% (5500/ 6307) | 87.6% (5354/ 6115) | 0.56 |
| Intrapartum antibiotic exposure | 10.8% (682/ 6290) | 10.8% (656/ 6092) | 0.89 |
| Neonatal antibiotic exposure | 10.7% (673/6307) | 7.8% (478/6115) | <0.0001 |
| Number of antibiotic prescriptions during follow-up* | 7 (4, 12) | 6 (3, 10) | <0.0001 |
| The data are presented as means (SD) for continuous variables and as percentage (proportion) for categorical variables. Student’s two-tailed t-test was used for continuous variables and Chi-squared test for categorical variables. **Median (quartiles Q1, Q3) and Wilcoxon rank-sum test were used because of normal distribution.* | | | |

| **Supplementary** **Table 2.** Breastfeeding rates of the children in the PEACHES cohort^a,b^. | | | |
| --- | --- | --- | --- |
|  | No infection and no neonatal antibiotics (N=572) | Infection and neonatal antibiotics (N=37) | *p* |
| Never breastfed | 39 (6.8%) | 3 (8.1%) | 0.73 |
| Breastfed at 1 month | 533 (93.2%) | 34 (91.9%) | 0.73 |
| Breastfed at 2 months | 496 (86.7%) | 34 (91.9%) | 0.46 |
| Breastfed at 3 months | 469 (82.1%) | 31 (83.8%) | 1.00 |
| Breastfed at 4 months | 449 (78.8%) | 30 (81.1%) | 0.84 |
| Breastfed at 5 months | 419 (73.5%) | 25 (67.6%) | 0.45 |
| Breastfed at 6 months | 391 (68.6%) | 22 (59.5%) | 0.28 |
| ^a^ Rates are presented for all children in the PEACHES cohort who fulfilled the inclusion criteria for analysis and had data of at least one breastfeeding occurrence and information on neonatal infection and antibiotic therapy.  ^b^ Dichotomous variables were assessed using the Fisher’s exact test. | | | |

| **Supplementary** **Table 3.** Clinical characteristics of the neonates included in the gut microbiome study. | | | |
| --- | --- | --- | --- |
|  | Neonatal antibiotics (N=13) | Control (N=20) | *p* |
| Gestational age (weeks) | 39^5/7^ (36^0/7^-42^1/7^) | 40^4/7^ (37^4/7^-42^0/7^) | 0.17 |
| Birth weight (grams) | 3708 (2810-4660) | 3729 (2930-4460) | 0.90 |
| Intrapartum antibiotic exposure | 71% (5/13) | 10% (2/20) | 0.052 |
| Vaginal delivery | 92% (12/13) | 100% (20/20) | 0.17 |
| Female | 31% (4/13) | 45% (9/20) | 0.41 |
| 5 min Apgar score | 7.9 (3-10) | 9.0 (8-10) | 0.10 |
| Breastfed at 1 month | 100% (13/13) | 100% (20/20) | NA |
| Breastfeeding duration (months) | 7.7 (1.5 to 13.0) | 10.5 (6.0-14.0) | 0.031 |
| Antibiotics before 6 months of age | 15% (2/13) | 5% (1/20) | 0.32 |
| Continuous data are expressed as means with range, and the differences between groups were assessed using Student’s two-tailed t test. Dichotomous data are expressed as percentages (proportions) and were assessed using the Chi square test. NA not applicable. | | | |

| **Supplementary** **Table 4.** Hotelling test results on the 3 first dimensions of the PCoA projection among groups | | |
| --- | --- | --- |
| Timepoint (month) | 3 PCoA dimensions P value |  |
| 1 | 0.002 |  |
| 6 | 0.003 |  |
| 12 | >0.05 |  |
| 24 | >0.05 |  |

| **Supplementary** **Table 5.** Metagenomic analysis sample information | | | | | | | | |
| --- | --- | --- | --- | --- | --- | --- | --- | --- |
| Sample | Baby | Age (months) | Control/Abx | # reads (raw) | # bps (raw) | # bps in scaffolds >= 500 bp | % of reads mapped to assembly | # species >= 0.1% |
| 104_CTR_1M | 104 | 1 | CTR | 27,255,538 | 3,048,490,679 | 42,168,529 | 98.68 | 19 |
| 126_CTR_1M | 126 | 1 | CTR | 27,436,708 | 2,704,057,331 | 26,537,329 | 99.22 | 11 |
| 126_CTR_6M | 126 | 6 | CTR | 19,552,656 | 1,924,583,937 | 31,676,468 | 97.76 | 16 |
| 128_CTR_1M | 128 | 1 | CTR | 19,087,812 | 1,882,462,171 | 13,345,768 | 98.64 | 6 |
| 128_CTR_6M | 128 | 6 | CTR | 21,249,252 | 2,093,497,953 | 44,003,659 | 97.21 | 21 |
| 13_CTR_1M | 13 | 1 | CTR | 31,323,492 | 3,113,344,228 | 36,535,975 | 98.4 | 15 |
| 13_CTR_6M | 13 | 6 | CTR | 15,724,876 | 1,821,780,314 | 24,510,972 | 95.49 | 17 |
| 135_CTR_1M | 135 | 1 | CTR | 21,813,766 | 2,164,597,856 | 11,208,704 | 99.32 | 6 |
| 139_CTR_1M | 139 | 1 | CTR | 49,758,574 | 5,743,483,187 | 15,510,806 | 95.61 | 5 |
| 142_CTR_6M | 142 | 6 | CTR | 15,091,622 | 1,499,351,446 | 22,484,164 | 98.93 | 8 |
| 15_ABX_1M | 15 | 1 | ABX | 13,854,074 | 1,451,391,519 | 24,402,659 | 94.78 | 12 |
| 15_ABX_6M | 15 | 6 | ABX | 27,280,609 | 3,060,212,469 | 36,659,134 | 95.19 | 13 |
| 157_ABX_1M | 157 | 1 | ABX | 52,738,236 | 6,074,658,129 | 24,887,402 | 95.68 | 12 |
| 165_ABX_6M | 165 | 6 | ABX | 13,835,151 | 1,601,573,754 | 67,265,903 | 92.71 | 32 |
| 166_ABX_1M | 166 | 1 | ABX | 14,206,158 | 1,654,818,160 | 27,287,133 | 96.28 | 12 |
| 166_ABX_6M | 166 | 6 | ABX | 41,065,702 | 4,242,709,634 | 74,970,475 | 97.38 | 23 |
| 179_CTR_6M | 179 | 6 | CTR | 11,002,326 | 1,092,263,236 | 19,392,960 | 96.69 | 14 |
| 182_ABX_6M | 182 | 6 | ABX | 35,025,777 | 3,999,836,943 | 36,641,137 | 95.32 | 15 |
| 183_ABX_1M | 183 | 1 | ABX | 18,023,026 | 2,131,535,284 | 15,706,291 | 96.81 | 13 |
| 184_CTR_1M | 184 | 1 | CTR | 20,058,642 | 1,987,914,876 | 16,233,759 | 97.8 | 11 |
| 184_CTR_6M | 184 | 6 | CTR | 53,466,603 | 5,961,513,660 | 43,372,254 | 95.3 | 14 |
| 193_ABX_6M | 193 | 6 | ABX | 37,799,693 | 4,190,740,176 | 47,149,951 | 95.36 | 15 |
| 20_CTR_6M | 20 | 6 | CTR | 22,479,101 | 2,543,061,606 | 48,997,667 | 94.02 | 24 |
| 21_CTR_6M | 21 | 6 | CTR | 16,699,452 | 1,887,414,687 | 16,563,639 | 94.81 | 10 |
| 68_CTR_6M | 68 | 6 | CTR | 33,369,070 | 3,885,283,530 | 51,837,678 | 94.51 | 18 |
| 86_ABX_1M | 86 | 1 | ABX | 14,355,072 | 1,426,798,963 | 20,352,885 | 97.22 | 16 |
| 86_ABX_6M | 86 | 6 | ABX | 37,252,284 | 3,688,793,852 | 60,501,777 | 97.01 | 22 |
|  |  |  |  |  |  |  |  |  |
| Reported number of reads refers to reads that passed trimming. | | | | | |  |  |  |

| **Supplementary** **Table 6.** Information about genomes recovered in the study. | | | | | | | | |
| --- | --- | --- | --- | --- | --- | --- | --- | --- |
| Sample | Genome name | Species | # scaffolds | Size | Completeness | Contamination | Present in other sample? | Metaphlan2 abundance |
| 104_CTR_1M | 104_CTR_1M_60_470.B_longum | B. longum | 47 | 2,253,538 | 100 | 0 |  | 59.55401 |
| 126_CTR_1M | 126_CTR_1M_60_30.B_longum | B. longum | 138 | 2,683,561 | 100 | 1.04 | Same as 126_CTR_6M_60_57.B_longum | 5.02798 |
| 126_CTR_6M | 126_CTR_6M_55_5.B_longum | B. longum | 82 | 80,384 | 6.18 | 0.86 | No | 14.24287 |
| 126_CTR_6M | 126_CTR_6M_60_57.B_longum | B. longum | 121 | 2,680,149 | 100 | 0.58 | Same as 126_CTR_1M_60_30.B_longum | 14.24287 |
| 128_CTR_1M | 128_CTR_1M_63_61.B_bifidum | B. bifidum | 72 | 2,237,613 | 99.31 | 0.46 | Same as 128_CTR_6M_63_36.B_bifidum | 7.72694 |
| 128_CTR_1M | 128_CTR_1M_59_629.B_breve | B. breve | 32 | 2,323,178 | 100 | 0 | Same as 128_CTR_6M_59_42.B_breve | 85.95728 |
| 128_CTR_6M | 128_CTR_6M_63_36.B_bifidum | B. bifidum | 61 | 2,235,764 | 99.31 | 0 | Same as 128_CTR_1M_63_61.B_bifidum | 10.66277 |
| 128_CTR_6M | 128_CTR_6M_59_42.B_breve | B. breve | 63 | 2,313,448 | 99.77 | 0 | Same as 128_CTR_1M_59_629.B_breve | 13.73136 |
| 128_CTR_6M | 128_CTR_6M_57_14.B_pseudocatenulatum | B. pseudocatenulatum | 161 | 2,134,790 | 99.09 | 2.57 |  | 1.6977 |
| 13_CTR_1M | 13_CTR_1M_59_170.B_breve | B. breve | 230 | 2,345,256 | 99.73 | 0.96 |  | 22.81031 |
| 13_CTR_1M | 13_CTR_1M_60_58.B_longum | B. longum | 216 | 2,267,392 | 94.89 | 0.96 |  | 7.63968 |
| 13_CTR_1M | 13_CTR_1M_63_26.B_bifidum | B. bifidum | 103 | 2,275,200 | 99.31 | 2.88 |  | 3.25749 |
| 13_CTR_1M | 13_CTR_1M_58_6.B_dentium | B. dentium | 1,250 | 2,182,660 | 76.91 | 4.75 |  | 0.57315 |
| 13_CTR_6M | 13_CTR_6M_63_11.B_bifidum | B. bifidum | 156 | 2,190,750 | 95.23 | 3.01 |  | 4.0731 |
| 13_CTR_6M | 13_CTR_6M_59_30.B_breve | B. breve | 109 | 2,226,565 | 93.03 | 2.09 |  | 13.24834 |
| 13_CTR_6M | 13_CTR_6M_60_16.B_longum | B. longum | 152 | 2,121,967 | 91.11 | 2.15 |  | 6.35087 |
| 13_CTR_6M | NA | B. dentium | NA | NA | NA | NA |  | 0.44405 |
| 139_CTR_1M | 139_CTR_1M_63_983.B_bifidum | B. bifidum | 26 | 2,204,467 | 99.08 | 0 |  | 86.1316 |
| 139_CTR_1M | 139_CTR_1M_56_415.B_pseudocatenulatum | B. pseudocatenulatum | 16 | 2,169,171 | 100 | 0.68 |  | 10.35922 |
| 142_CTR_6M | 142_CTR_6M_60_10.B_longum | B. longum | 360 | 2,458,933 | 98.92 | 5.12 |  | 5.9558 |
| 15_ABX_1M | 15_ABX_1M_60_20.B_longum | B. longum | 51 | 2,171,246 | 96.34 | 0.32 |  | 13.52892 |
| 15_ABX_6M | 15_ABX_6M_63_14.B_bifidum | B. bifidum | 53 | 2,107,053 | 95.62 | 0.58 |  | 2.38693 |
| 15_ABX_6M | 15_ABX_6M_59_411.B_breve | B. breve | 37 | 2,289,829 | 95.99 | 1.84 |  | 84.7879 |
| 15_ABX_6M | 15_ABX_6M_60_31.B_longum | B. longum | 116 | 1,977,940 | 86.44 | 0.25 |  | 5.17153 |
| 165_ABX_6M | 165_ABX_6M_59_4.B_adolescentis | B. adolescentis | 94 | 255,422 | 4.17 | 0 |  | 1.46765 |
| 165_ABX_6M | 165_ABX_6M_59_51.B_breve | B. breve | 97 | 2,051,765 | 81.99 | 0.98 |  | 20.92734 |
| 165_ABX_6M | 165_ABX_6M_60_38.B_longum | B. longum | 114 | 2,186,260 | 92.91 | 2.09 |  | 13.31265 |
| 165_ABX_6M | 165_ABX_6M_56_33.B_pseudocatenulatum | B. pseudocatenulatum | 33 | 2,077,285 | 91.59 | 0.38 |  | 6.30968 |
| 166_ABX_6M | 166_ABX_6M_58_66.B_breve | B. breve | 116 | 1,786,283 | 82.32 | 1.27 |  | 8.73569 |
| 166_ABX_6M | 166_ABX_6M_59_206.B_longum | B. longum | 190 | 1,093,504 | 21 | 0 |  | 40.54609 |
| 166_ABX_6M | 166_ABX_6M_60_431.B_longum | B. longum | 273 | 2,183,475 | 78.34 | 2.22 |  | 40.54609 |
| 179_CTR_6M | 179_CTR_6M_60_119.B_longum | B. longum | 242 | 2,322,072 | 100 | 1.65 |  | 58.18272 |
| 179_CTR_6M | 179_CTR_6M_63_9.B_bifidum | B. bifidum | 589 | 2,221,156 | 94.37 | 6.81 |  | 4.36336 |
| 179_CTR_6M | 179_CTR_6M_57_21.B_longum | B. longum | 121 | 557,897 | 0 | 0 |  | 58.18272 |
| 182_ABX_6M | 182_ABX_6M_59_41.B_breve | B. breve | 125 | 2,007,736 | 90.26 | 0.69 |  | 10.06898 |
| 182_ABX_6M | 182_ABX_6M_60_296.B_longum | B. longum | 108 | 2,567,290 | 96.49 | 2.49 |  | 1.73043 |
| 184_CTR_1M | 184_CTR_1M_60_247.B_longum | B. longum | 57 | 2,585,388 | 99.77 | 9.79 |  | 41.16457 |
| 184_CTR_1M | 184_CTR_1M_63_198.B_bifidum | B. bifidum | 49 | 1,987,643 | 90.32 | 1.38 |  | 32.22095 |
| 184_CTR_6M | 184_CTR_6M_63_270.B_bifidum | B. bifidum | 29 | 2,131,794 | 99.08 | 0.46 |  | 18.36808 |
| 184_CTR_6M | 184_CTR_6M_59_30.B_dentium | B. dentium | 52 | 2,264,988 | 85.11 | 0.91 |  | 1.32606 |
| 184_CTR_6M | 184_CTR_6M_60_761.B_longum | B. longum | 34 | 2,296,474 | 99.77 | 0.46 |  | 52.50827 |
| 193_ABX_6M | 193_ABX_6M_58_31.B_dentium | B. dentium | 22 | 2,267,060 | 90.76 | 0.45 |  | 3.59905 |
| 193_ABX_6M | 193_ABX_6M_60_238.B_longum | B. longum | 14 | 1,858,493 | 80.55 | 0.46 |  | 33.56637 |
| 20_CTR_6M | 20_CTR_6M_60_150.B_longum | B. longum | 68 | 2,141,707 | 91.01 | 0.61 |  | 34.24537 |
| 20_CTR_6M | 20_CTR_6M_56_136.B_pseudocatenulatum | B. pseudocatenulatum | 16 | 1,946,461 | 76.73 | 0 |  | 20.98682 |
| 21_CTR_6M | 21_CTR_6M_63_133.B_bifidum | B. bifidum | 33 | 2,175,665 | 99.08 | 0 |  | 27.73302 |
| 21_CTR_6M | 21_CTR_6M_59_185.B_breve | B. breve | 67 | 2,465,640 | 99.68 | 2.79 |  | 44.71957 |
| 68_CTR_6M | 68_CTR_6M_61_290.B_bifidum | B. bifidum | 19 | 2,137,251 | 97.47 | 1 |  | 31.56724 |
| 68_CTR_6M | 68_CTR_6M_59_242.B_breve | B. breve | 132 | 2,371,135 | 96.31 | 7.97 |  | 28.40702 |
| 68_CTR_6M | 68_CTR_6M_60_54.B_longum | B. longum | 132 | 2,166,607 | 96.58 | 3.15 |  | 5.3631 |
| 86_ABX_6M | 86_ABX_6M_60_467.B_longum | B. longum | 58 | 2,282,026 | 100 | 0.12 |  | 49.99088 |
| 86_ABX_6M | 86_ABX_6M_58_194.B_dentium | B. dentium | 51 | 2,555,581 | 97.61 | 1.21 |  | 16.89689 |
| 86_ABX_6M | 86_ABX_6M_59_234.B_adolescentis | B. adolescentis | 46 | 2,282,883 | 97.46 | 1.21 |  | 17.07824 |
| Completeness and contamination were determined using CheckM, abundance is based on Metaphlan2. | | | | | | | | |

| **Supplementary** **Table 7**. Summary of *Bifidobacterium* species presence/absence in the different samples | | | | | | | | | |  |
| --- | --- | --- | --- | --- | --- | --- | --- | --- | --- | --- |
| Sample | Age | Status | B. adolescentis | B. bifidum | B. breve | B. dentium | B. longum | B. pseudocatenulatum |  |  |
| 104_CTR_1M | 1 | CTR | 0 | 0 | 0 | 0 | 1 | 0 |  |  |
| 126_CTR_1M | 1 | CTR | 0 | 0 | 0 | 0 | 1 | 0 |  |  |
| 126_CTR_6M | 6 | CTR | 0 | 0 | 0 | 0 | 1 | 0 |  |  |
| 128_CTR_1M | 1 | CTR | 0 | 1 | 1 | 0 | 0 | 0 |  |  |
| 128_CTR_6M | 6 | CTR | 0 | 1 | 1 | 0 | 0 | 1 |  |  |
| 13_CTR_1M | 1 | CTR | 0 | 1 | 1 | 1 | 1 | 0 |  |  |
| 13_CTR_6M | 6 | CTR | 0 | 1 | 1 | 1 | 1 | 0 |  |  |
| 135_CTR_1M | 1 | CTR | 0 | 0 | 0 | 0 | 0 | 0 |  |  |
| 139_CTR_1M | 1 | CTR | 0 | 1 | 0 | 0 | 0 | 1 |  |  |
| 142_CTR_6M | 6 | CTR | 0 | 0 | 0 | 0 | 1 | 0 |  |  |
| 15_ABX_1M | 1 | ABX | 0 | 0 | 0 | 0 | 1 | 0 |  |  |
| 15_ABX_6M | 6 | ABX | 0 | 1 | 1 | 0 | 1 | 0 |  |  |
| 157_ABX_1M | 1 | ABX | 0 | 0 | 0 | 0 | 0 | 0 |  |  |
| 165_ABX_6M | 6 | ABX | 1 | 0 | 1 | 0 | 1 | 1 |  |  |
| 166_ABX_1M | 1 | ABX | 0 | 0 | 0 | 0 | 0 | 0 |  |  |
| 166_ABX_6M | 6 | ABX | 0 | 0 | 1 | 0 | 1 | 0 |  |  |
| 179_CTR_6M | 6 | CTR | 0 | 1 | 0 | 0 | 1 | 0 |  |  |
| 182_ABX_6M | 6 | ABX | 0 | 0 | 1 | 0 | 1 | 0 |  |  |
| 183_ABX_1M | 1 | ABX | 0 | 0 | 0 | 0 | 0 | 0 |  |  |
| 184_CTR_1M | 1 | CTR | 0 | 1 | 0 | 1 | 1 | 0 |  |  |
| 184_CTR_6M | 6 | CTR | 0 | 1 | 0 | 1 | 1 | 0 |  |  |
| 193_ABX_6M | 6 | ABX | 0 | 0 | 0 | 1 | 1 | 0 |  |  |
| 20_CTR_6M | 6 | CTR | 0 | 0 | 0 | 0 | 1 | 1 |  |  |
| 21_CTR_6M | 6 | CTR | 0 | 1 | 1 | 0 | 0 | 0 |  |  |
| 68_CTR_6M | 6 | CTR | 1 | 1 | 1 | 0 | 1 | 0 |  |  |
| 86_ABX_1M | 1 | ABX | 0 | 0 | 0 | 0 | 0 | 0 |  |  |
| 86_ABX_6M | 6 | ABX | 1 | 0 | 0 | 1 | 1 | 0 |  |  |

| **Supplementary** **Table 8.** Breakdown of species presence in the different groups. | | | | | | | | |
| --- | --- | --- | --- | --- | --- | --- | --- | --- |
| Species | CTR/1M (n=7) | ABX/1M (n=5) | CTR/6M (n=9) | ABX/6M (n=6) | CTR/1M (Frac) | ABX/1M (Frac) | CTR/6M (Frac) | ABX/6M (Frac) |
| k__Bacteria\|p__Actinobacteria\|c__Actinobacteria\|o__Bifidobacteriales\|f__Bifidobacteriaceae\|g__Bifidobacterium\|s__Bifidobacterium_bifidum | 4 | 0 | 6 | 1 | 0.57 | 0.00 | 0.67 | 0.17 |
| k__Bacteria\|p__Proteobacteria\|c__Gammaproteobacteria\|o__Enterobacteriales\|f__Enterobacteriaceae\|g__Escherichia\|s__Escherichia_coli | 5 | 1 | 9 | 4 | 0.71 | 0.20 | 1.00 | 0.67 |
| k__Bacteria\|p__Actinobacteria\|c__Actinobacteria\|o__Bifidobacteriales\|f__Bifidobacteriaceae\|g__Bifidobacterium\|s__Bifidobacterium_longum | 4 | 1 | 7 | 6 | 0.57 | 0.20 | 0.78 | 1.00 |
| k__Bacteria\|p__Firmicutes\|c__Negativicutes\|o__Selenomonadales\|f__Veillonellaceae\|g__Veillonella\|s__Veillonella_parvula | 4 | 1 | 8 | 5 | 0.57 | 0.20 | 0.89 | 0.83 |
| k__Bacteria\|p__Actinobacteria\|c__Actinobacteria\|o__Actinomycetales\|f__Micrococcaceae\|g__Rothia\|s__Rothia_mucilaginosa | 2 | 0 | 1 | 2 | 0.29 | 0.00 | 0.11 | 0.33 |
| k__Bacteria\|p__Actinobacteria\|c__Actinobacteria\|o__Bifidobacteriales\|f__Bifidobacteriaceae\|g__Bifidobacterium\|s__Bifidobacterium_breve | 2 | 0 | 4 | 4 | 0.29 | 0.00 | 0.44 | 0.67 |
| k__Bacteria\|p__Actinobacteria\|c__Actinobacteria\|o__Bifidobacteriales\|f__Bifidobacteriaceae\|g__Bifidobacterium\|s__Bifidobacterium_dentium | 2 | 0 | 2 | 2 | 0.29 | 0.00 | 0.22 | 0.33 |
| k__Bacteria\|p__Actinobacteria\|c__Actinobacteria\|o__Coriobacteriales\|f__Coriobacteriaceae\|g__Collinsella\|s__Collinsella_aerofaciens | 2 | 0 | 2 | 2 | 0.29 | 0.00 | 0.22 | 0.33 |
| k__Bacteria\|p__Firmicutes\|c__Bacilli\|o__Bacillales\|f__Staphylococcaceae\|g__Staphylococcus\|s__Staphylococcus_hominis | 2 | 0 | 0 | 0 | 0.29 | 0.00 | 0.00 | 0.00 |
| k__Bacteria\|p__Firmicutes\|c__Bacilli\|o__Lactobacillales\|f__Streptococcaceae\|g__Streptococcus\|s__Streptococcus_parasanguinis | 3 | 1 | 3 | 2 | 0.43 | 0.20 | 0.33 | 0.33 |
| k__Bacteria\|p__Firmicutes\|c__Bacilli\|o__Lactobacillales\|f__Streptococcaceae\|g__Streptococcus\|s__Streptococcus_vestibularis | 3 | 1 | 1 | 0 | 0.43 | 0.20 | 0.11 | 0.00 |
| k__Bacteria\|p__Actinobacteria\|c__Actinobacteria\|o__Actinomycetales\|f__Actinomycetaceae\|g__Actinomyces\|s__Actinomyces_neuii | 1 | 0 | 0 | 0 | 0.14 | 0.00 | 0.00 | 0.00 |
| k__Bacteria\|p__Actinobacteria\|c__Actinobacteria\|o__Bifidobacteriales\|f__Bifidobacteriaceae\|g__Bifidobacterium\|s__Bifidobacterium_pseudocatenulatum | 1 | 0 | 2 | 1 | 0.14 | 0.00 | 0.22 | 0.17 |
| k__Bacteria\|p__Actinobacteria\|c__Actinobacteria\|o__Bifidobacteriales\|f__Bifidobacteriaceae\|g__Parascardovia\|s__Parascardovia_denticolens | 1 | 0 | 0 | 0 | 0.14 | 0.00 | 0.00 | 0.00 |
| k__Bacteria\|p__Bacteroidetes\|c__Bacteroidia\|o__Bacteroidales\|f__Bacteroidaceae\|g__Bacteroides\|s__Bacteroides_caccae | 1 | 0 | 1 | 0 | 0.14 | 0.00 | 0.11 | 0.00 |
| k__Bacteria\|p__Bacteroidetes\|c__Bacteroidia\|o__Bacteroidales\|f__Bacteroidaceae\|g__Bacteroides\|s__Bacteroides_dorei | 1 | 0 | 4 | 0 | 0.14 | 0.00 | 0.44 | 0.00 |
| k__Bacteria\|p__Bacteroidetes\|c__Bacteroidia\|o__Bacteroidales\|f__Bacteroidaceae\|g__Bacteroides\|s__Bacteroides_faecis | 1 | 0 | 2 | 0 | 0.14 | 0.00 | 0.22 | 0.00 |
| k__Bacteria\|p__Bacteroidetes\|c__Bacteroidia\|o__Bacteroidales\|f__Bacteroidaceae\|g__Bacteroides\|s__Bacteroides_sp_3_1_19 | 1 | 0 | 1 | 0 | 0.14 | 0.00 | 0.11 | 0.00 |
| k__Bacteria\|p__Bacteroidetes\|c__Bacteroidia\|o__Bacteroidales\|f__Porphyromonadaceae\|g__Parabacteroides\|s__Parabacteroides_merdae | 1 | 0 | 0 | 0 | 0.14 | 0.00 | 0.00 | 0.00 |
| k__Bacteria\|p__Bacteroidetes\|c__Bacteroidia\|o__Bacteroidales\|f__Porphyromonadaceae\|g__Parabacteroides\|s__Parabacteroides_unclassified | 1 | 0 | 1 | 1 | 0.14 | 0.00 | 0.11 | 0.17 |
| k__Bacteria\|p__Firmicutes\|c__Bacilli\|o__Lactobacillales\|f__Lactobacillaceae\|g__Lactobacillus\|s__Lactobacillus_crispatus | 1 | 0 | 0 | 0 | 0.14 | 0.00 | 0.00 | 0.00 |
| k__Bacteria\|p__Firmicutes\|c__Bacilli\|o__Lactobacillales\|f__Lactobacillaceae\|g__Lactobacillus\|s__Lactobacillus_fermentum | 1 | 0 | 2 | 0 | 0.14 | 0.00 | 0.22 | 0.00 |
| k__Bacteria\|p__Firmicutes\|c__Bacilli\|o__Lactobacillales\|f__Lactobacillaceae\|g__Lactobacillus\|s__Lactobacillus_gasseri | 1 | 0 | 0 | 0 | 0.14 | 0.00 | 0.00 | 0.00 |
| k__Bacteria\|p__Firmicutes\|c__Bacilli\|o__Lactobacillales\|f__Streptococcaceae\|g__Streptococcus\|s__Streptococcus_mitis_oralis_pneumoniae | 1 | 0 | 1 | 0 | 0.14 | 0.00 | 0.11 | 0.00 |
| k__Bacteria\|p__Firmicutes\|c__Clostridia\|o__Clostridiales\|f__Lachnospiraceae\|g__Blautia\|s__Ruminococcus_gnavus | 1 | 0 | 2 | 3 | 0.14 | 0.00 | 0.22 | 0.50 |
| k__Bacteria\|p__Firmicutes\|c__Negativicutes\|o__Selenomonadales\|f__Veillonellaceae\|g__Veillonella\|s__Veillonella_atypica | 2 | 1 | 4 | 3 | 0.29 | 0.20 | 0.44 | 0.50 |
| k__Bacteria\|p__Proteobacteria\|c__Gammaproteobacteria\|o__Enterobacteriales\|f__Enterobacteriaceae\|g__Klebsiella\|s__Klebsiella_unclassified | 1 | 0 | 1 | 1 | 0.14 | 0.00 | 0.11 | 0.17 |
| k__Bacteria\|p__Proteobacteria\|c__Gammaproteobacteria\|o__Pasteurellales\|f__Pasteurellaceae\|g__Haemophilus\|s__Haemophilus_sputorum | 1 | 0 | 0 | 0 | 0.14 | 0.00 | 0.00 | 0.00 |
| k__Bacteria\|p__Actinobacteria\|c__Actinobacteria\|o__Actinomycetales\|f__Actinomycetaceae\|g__Actinomyces\|s__Actinomyces_urogenitalis | 0 | 0 | 1 | 0 | 0.00 | 0.00 | 0.11 | 0.00 |
| k__Bacteria\|p__Actinobacteria\|c__Actinobacteria\|o__Bifidobacteriales\|f__Bifidobacteriaceae\|g__Bifidobacterium\|s__Bifidobacterium_adolescentis | 0 | 0 | 1 | 2 | 0.00 | 0.00 | 0.11 | 0.33 |
| k__Bacteria\|p__Actinobacteria\|c__Actinobacteria\|o__Coriobacteriales\|f__Coriobacteriaceae\|g__Eggerthella\|s__Eggerthella_lenta | 0 | 0 | 0 | 1 | 0.00 | 0.00 | 0.00 | 0.17 |
| k__Bacteria\|p__Actinobacteria\|c__Actinobacteria\|o__Coriobacteriales\|f__Coriobacteriaceae\|g__Eggerthella\|s__Eggerthella_unclassified | 0 | 0 | 5 | 3 | 0.00 | 0.00 | 0.56 | 0.50 |
| k__Bacteria\|p__Bacteroidetes\|c__Bacteroidia\|o__Bacteroidales\|f__Bacteroidaceae\|g__Bacteroides\|s__Bacteroides_cellulosilyticus | 0 | 0 | 1 | 0 | 0.00 | 0.00 | 0.11 | 0.00 |
| k__Bacteria\|p__Bacteroidetes\|c__Bacteroidia\|o__Bacteroidales\|f__Bacteroidaceae\|g__Bacteroides\|s__Bacteroides_fragilis | 1 | 1 | 2 | 1 | 0.14 | 0.20 | 0.22 | 0.17 |
| k__Bacteria\|p__Bacteroidetes\|c__Bacteroidia\|o__Bacteroidales\|f__Bacteroidaceae\|g__Bacteroides\|s__Bacteroides_sp_3_2_5 | 0 | 0 | 0 | 1 | 0.00 | 0.00 | 0.00 | 0.17 |
| k__Bacteria\|p__Bacteroidetes\|c__Bacteroidia\|o__Bacteroidales\|f__Bacteroidaceae\|g__Bacteroides\|s__Bacteroides_uniformis | 1 | 1 | 3 | 2 | 0.14 | 0.20 | 0.33 | 0.33 |
| k__Bacteria\|p__Bacteroidetes\|c__Bacteroidia\|o__Bacteroidales\|f__Bacteroidaceae\|g__Bacteroides\|s__Bacteroides_vulgatus | 0 | 0 | 1 | 2 | 0.00 | 0.00 | 0.11 | 0.33 |
| k__Bacteria\|p__Bacteroidetes\|c__Bacteroidia\|o__Bacteroidales\|f__Bacteroidaceae\|g__Bacteroides\|s__Bacteroides_xylanisolvens | 0 | 0 | 0 | 1 | 0.00 | 0.00 | 0.00 | 0.17 |
| k__Bacteria\|p__Bacteroidetes\|c__Bacteroidia\|o__Bacteroidales\|f__Porphyromonadaceae\|g__Barnesiella\|s__Barnesiella_intestinihominis | 0 | 0 | 0 | 1 | 0.00 | 0.00 | 0.00 | 0.17 |
| k__Bacteria\|p__Firmicutes\|c__Bacilli\|o__Bacillales\|f__Staphylococcaceae\|g__Staphylococcus\|s__Staphylococcus_epidermidis | 3 | 3 | 0 | 0 | 0.43 | 0.60 | 0.00 | 0.00 |
| k__Bacteria\|p__Firmicutes\|c__Bacilli\|o__Lactobacillales\|f__Enterococcaceae\|g__Enterococcus\|s__Enterococcus_casseliflavus | 0 | 0 | 1 | 1 | 0.00 | 0.00 | 0.11 | 0.17 |
| k__Bacteria\|p__Firmicutes\|c__Bacilli\|o__Lactobacillales\|f__Enterococcaceae\|g__Enterococcus\|s__Enterococcus_durans | 0 | 0 | 0 | 1 | 0.00 | 0.00 | 0.00 | 0.17 |
| k__Bacteria\|p__Firmicutes\|c__Bacilli\|o__Lactobacillales\|f__Lactobacillaceae\|g__Lactobacillus\|s__Lactobacillus_casei_paracasei | 0 | 0 | 2 | 1 | 0.00 | 0.00 | 0.22 | 0.17 |
| k__Bacteria\|p__Firmicutes\|c__Bacilli\|o__Lactobacillales\|f__Lactobacillaceae\|g__Lactobacillus\|s__Lactobacillus_rhamnosus | 2 | 2 | 5 | 2 | 0.29 | 0.40 | 0.56 | 0.33 |
| k__Bacteria\|p__Firmicutes\|c__Bacilli\|o__Lactobacillales\|f__Streptococcaceae\|g__Streptococcus\|s__Streptococcus_lutetiensis | 0 | 0 | 1 | 0 | 0.00 | 0.00 | 0.11 | 0.00 |
| k__Bacteria\|p__Firmicutes\|c__Bacilli\|o__Lactobacillales\|f__Streptococcaceae\|g__Streptococcus\|s__Streptococcus_peroris | 0 | 0 | 1 | 1 | 0.00 | 0.00 | 0.11 | 0.17 |
| k__Bacteria\|p__Firmicutes\|c__Bacilli\|o__Lactobacillales\|f__Streptococcaceae\|g__Streptococcus\|s__Streptococcus_salivarius | 5 | 5 | 4 | 1 | 0.71 | 1.00 | 0.44 | 0.17 |
| k__Bacteria\|p__Firmicutes\|c__Bacilli\|o__Lactobacillales\|f__Streptococcaceae\|g__Streptococcus\|s__Streptococcus_sp_C150 | 0 | 0 | 0 | 1 | 0.00 | 0.00 | 0.00 | 0.17 |
| k__Bacteria\|p__Firmicutes\|c__Clostridia\|o__Clostridiales\|f__Clostridiales_noname\|g__Flavonifractor\|s__Flavonifractor_plautii | 0 | 0 | 0 | 1 | 0.00 | 0.00 | 0.00 | 0.17 |
| k__Bacteria\|p__Firmicutes\|c__Clostridia\|o__Clostridiales\|f__Eubacteriaceae\|g__Eubacterium\|s__Eubacterium_hallii | 0 | 0 | 1 | 0 | 0.00 | 0.00 | 0.11 | 0.00 |
| k__Bacteria\|p__Firmicutes\|c__Clostridia\|o__Clostridiales\|f__Eubacteriaceae\|g__Eubacterium\|s__Eubacterium_limosum | 0 | 0 | 0 | 1 | 0.00 | 0.00 | 0.00 | 0.17 |
| k__Bacteria\|p__Firmicutes\|c__Clostridia\|o__Clostridiales\|f__Lachnospiraceae\|g__Blautia\|s__Blautia_producta | 0 | 0 | 0 | 2 | 0.00 | 0.00 | 0.00 | 0.33 |
| k__Bacteria\|p__Firmicutes\|c__Clostridia\|o__Clostridiales\|f__Lachnospiraceae\|g__Blautia\|s__Ruminococcus_torques | 0 | 0 | 1 | 2 | 0.00 | 0.00 | 0.11 | 0.33 |
| k__Bacteria\|p__Firmicutes\|c__Clostridia\|o__Clostridiales\|f__Lachnospiraceae\|g__Dorea\|s__Dorea_formicigenerans | 0 | 0 | 1 | 0 | 0.00 | 0.00 | 0.11 | 0.00 |
| k__Bacteria\|p__Firmicutes\|c__Clostridia\|o__Clostridiales\|f__Lachnospiraceae\|g__Lachnospiraceae_noname\|s__Lachnospiraceae_bacterium_2_1_58FAA | 0 | 0 | 1 | 1 | 0.00 | 0.00 | 0.11 | 0.17 |
| k__Bacteria\|p__Firmicutes\|c__Clostridia\|o__Clostridiales\|f__Lachnospiraceae\|g__Lachnospiraceae_noname\|s__Lachnospiraceae_bacterium_3_1_57FAA_CT1 | 0 | 0 | 0 | 1 | 0.00 | 0.00 | 0.00 | 0.17 |
| k__Bacteria\|p__Firmicutes\|c__Clostridia\|o__Clostridiales\|f__Lachnospiraceae\|g__Lachnospiraceae_noname\|s__Lachnospiraceae_bacterium_5_1_63FAA | 0 | 0 | 1 | 1 | 0.00 | 0.00 | 0.11 | 0.17 |
| k__Bacteria\|p__Firmicutes\|c__Clostridia\|o__Clostridiales\|f__Lachnospiraceae\|g__Roseburia\|s__Roseburia_hominis | 0 | 0 | 0 | 1 | 0.00 | 0.00 | 0.00 | 0.17 |
| k__Bacteria\|p__Firmicutes\|c__Clostridia\|o__Clostridiales\|f__Lachnospiraceae\|g__Roseburia\|s__Roseburia_intestinalis | 0 | 0 | 0 | 1 | 0.00 | 0.00 | 0.00 | 0.17 |
| k__Bacteria\|p__Firmicutes\|c__Clostridia\|o__Clostridiales\|f__Peptostreptococcaceae\|g__Peptostreptococcaceae_noname\|s__Clostridium_bartlettii | 0 | 0 | 2 | 5 | 0.00 | 0.00 | 0.22 | 0.83 |
| k__Bacteria\|p__Firmicutes\|c__Clostridia\|o__Clostridiales\|f__Peptostreptococcaceae\|g__Peptostreptococcaceae_noname\|s__Clostridium_difficile | 0 | 0 | 2 | 2 | 0.00 | 0.00 | 0.22 | 0.33 |
| k__Bacteria\|p__Firmicutes\|c__Clostridia\|o__Clostridiales\|f__Peptostreptococcaceae\|g__Peptostreptococcaceae_noname\|s__Peptostreptococcaceae_noname_unclassified | 0 | 0 | 1 | 0 | 0.00 | 0.00 | 0.11 | 0.00 |
| k__Bacteria\|p__Firmicutes\|c__Clostridia\|o__Clostridiales\|f__Ruminococcaceae\|g__Faecalibacterium\|s__Faecalibacterium_prausnitzii | 0 | 0 | 0 | 1 | 0.00 | 0.00 | 0.00 | 0.17 |
| k__Bacteria\|p__Firmicutes\|c__Erysipelotrichia\|o__Erysipelotrichales\|f__Erysipelotrichaceae\|g__Coprobacillus\|s__Coprobacillus_unclassified | 0 | 0 | 1 | 2 | 0.00 | 0.00 | 0.11 | 0.33 |
| k__Bacteria\|p__Firmicutes\|c__Erysipelotrichia\|o__Erysipelotrichales\|f__Erysipelotrichaceae\|g__Erysipelotrichaceae_noname\|s__Clostridium_innocuum | 0 | 0 | 1 | 0 | 0.00 | 0.00 | 0.11 | 0.00 |
| k__Bacteria\|p__Firmicutes\|c__Erysipelotrichia\|o__Erysipelotrichales\|f__Erysipelotrichaceae\|g__Erysipelotrichaceae_noname\|s__Clostridium_ramosum | 0 | 0 | 1 | 3 | 0.00 | 0.00 | 0.11 | 0.50 |
| k__Bacteria\|p__Firmicutes\|c__Negativicutes\|o__Selenomonadales\|f__Veillonellaceae\|g__Megasphaera\|s__Megasphaera_micronuciformis | 0 | 0 | 2 | 0 | 0.00 | 0.00 | 0.22 | 0.00 |
| k__Bacteria\|p__Firmicutes\|c__Negativicutes\|o__Selenomonadales\|f__Veillonellaceae\|g__Veillonella\|s__Veillonella_unclassified | 2 | 2 | 3 | 4 | 0.29 | 0.40 | 0.33 | 0.67 |
| k__Bacteria\|p__Proteobacteria\|c__Gammaproteobacteria\|o__Enterobacteriales\|f__Enterobacteriaceae\|g__Klebsiella\|s__Klebsiella_oxytoca | 1 | 1 | 2 | 5 | 0.14 | 0.20 | 0.22 | 0.83 |
| k__Bacteria\|p__Proteobacteria\|c__Gammaproteobacteria\|o__Enterobacteriales\|f__Enterobacteriaceae\|g__Raoultella\|s__Raoultella_ornithinolytica | 0 | 0 | 1 | 0 | 0.00 | 0.00 | 0.11 | 0.00 |
| k__Bacteria\|p__Proteobacteria\|c__Gammaproteobacteria\|o__Pasteurellales\|f__Pasteurellaceae\|g__Haemophilus\|s__Haemophilus_parainfluenzae | 2 | 2 | 5 | 3 | 0.29 | 0.40 | 0.56 | 0.50 |
| k__Viruses\|p__Viruses_noname\|c__Viruses_noname\|o__Caudovirales\|f__Siphoviridae\|g__Lambdalikevirus\|s__Enterobacteria_phage_mEpX1 | 0 | 0 | 1 | 1 | 0.00 | 0.00 | 0.11 | 0.17 |
| k__Viruses\|p__Viruses_noname\|c__Viruses_noname\|o__Caudovirales\|f__Siphoviridae\|g__Siphoviridae_noname\|s__Propionibacterium_phage_PAS50 | 0 | 0 | 0 | 1 | 0.00 | 0.00 | 0.00 | 0.17 |
| k__Viruses\|p__Viruses_noname\|c__Viruses_noname\|o__Viruses_noname\|f__Partitiviridae\|g__Alphacryptovirus\|s__Vicia_cryptic_virus | 0 | 0 | 3 | 2 | 0.00 | 0.00 | 0.33 | 0.33 |
| k__Viruses\|p__Viruses_noname\|c__Viruses_noname\|o__Viruses_noname\|f__Viruses_noname\|g__Viruses_noname\|s__Enterococcus_phage_EF62phi | 0 | 0 | 1 | 0 | 0.00 | 0.00 | 0.11 | 0.00 |
| k__Bacteria\|p__Actinobacteria\|c__Actinobacteria\|o__Actinomycetales\|f__Actinomycetaceae\|g__Actinomyces\|s__Actinomyces_graevenitzii | 0 | 1 | 0 | 0 | 0.00 | 0.20 | 0.00 | 0.00 |
| k__Bacteria\|p__Actinobacteria\|c__Actinobacteria\|o__Actinomycetales\|f__Actinomycetaceae\|g__Varibaculum\|s__Varibaculum_cambriense | 0 | 1 | 0 | 0 | 0.00 | 0.20 | 0.00 | 0.00 |
| k__Bacteria\|p__Actinobacteria\|c__Actinobacteria\|o__Bifidobacteriales\|f__Bifidobacteriaceae\|g__Scardovia\|s__Scardovia_wiggsiae | 0 | 1 | 0 | 0 | 0.00 | 0.20 | 0.00 | 0.00 |
| k__Bacteria\|p__Bacteroidetes\|c__Bacteroidia\|o__Bacteroidales\|f__Bacteroidaceae\|g__Bacteroides\|s__Bacteroides_massiliensis | 0 | 1 | 0 | 0 | 0.00 | 0.20 | 0.00 | 0.00 |
| k__Bacteria\|p__Bacteroidetes\|c__Bacteroidia\|o__Bacteroidales\|f__Bacteroidaceae\|g__Bacteroides\|s__Bacteroides_thetaiotaomicron | 0 | 1 | 0 | 0 | 0.00 | 0.20 | 0.00 | 0.00 |
| k__Bacteria\|p__Firmicutes\|c__Bacilli\|o__Lactobacillales\|f__Enterococcaceae\|g__Enterococcus\|s__Enterococcus_faecalis | 2 | 3 | 5 | 3 | 0.29 | 0.60 | 0.56 | 0.50 |
| k__Bacteria\|p__Firmicutes\|c__Bacilli\|o__Lactobacillales\|f__Enterococcaceae\|g__Enterococcus\|s__Enterococcus_faecium | 0 | 1 | 1 | 2 | 0.00 | 0.20 | 0.11 | 0.33 |
| k__Bacteria\|p__Firmicutes\|c__Clostridia\|o__Clostridiales\|f__Clostridiaceae\|g__Clostridium\|s__Clostridium_clostridioforme | 0 | 1 | 1 | 0 | 0.00 | 0.20 | 0.11 | 0.00 |
| k__Bacteria\|p__Firmicutes\|c__Clostridia\|o__Clostridiales\|f__Clostridiaceae\|g__Clostridium\|s__Clostridium_perfringens | 0 | 1 | 0 | 0 | 0.00 | 0.20 | 0.00 | 0.00 |
| k__Bacteria\|p__Firmicutes\|c__Clostridia\|o__Clostridiales\|f__Lachnospiraceae\|g__Anaerostipes\|s__Anaerostipes_unclassified | 0 | 1 | 0 | 0 | 0.00 | 0.20 | 0.00 | 0.00 |
| k__Bacteria\|p__Proteobacteria\|c__Gammaproteobacteria\|o__Enterobacteriales\|f__Enterobacteriaceae\|g__Citrobacter\|s__Citrobacter_freundii | 0 | 1 | 1 | 1 | 0.00 | 0.20 | 0.11 | 0.17 |
| k__Bacteria\|p__Proteobacteria\|c__Gammaproteobacteria\|o__Enterobacteriales\|f__Enterobacteriaceae\|g__Citrobacter\|s__Citrobacter_koseri | 0 | 1 | 0 | 0 | 0.00 | 0.20 | 0.00 | 0.00 |
| k__Bacteria\|p__Proteobacteria\|c__Gammaproteobacteria\|o__Enterobacteriales\|f__Enterobacteriaceae\|g__Citrobacter\|s__Citrobacter_unclassified | 0 | 1 | 1 | 1 | 0.00 | 0.20 | 0.11 | 0.17 |
| k__Bacteria\|p__Proteobacteria\|c__Gammaproteobacteria\|o__Enterobacteriales\|f__Enterobacteriaceae\|g__Escherichia\|s__Escherichia_unclassified | 3 | 4 | 7 | 4 | 0.43 | 0.80 | 0.78 | 0.67 |
| k__Viruses\|p__Viruses_noname\|c__Viruses_noname\|o__Caudovirales\|f__Podoviridae\|g__Epsilon15likevirus\|s__Epsilon15likevirus_unclassified | 0 | 1 | 0 | 0 | 0.00 | 0.20 | 0.00 | 0.00 |
| k__Bacteria\|p__Firmicutes\|c__Negativicutes\|o__Selenomonadales\|f__Veillonellaceae\|g__Veillonella\|s__Veillonella_dispar | 0 | 2 | 2 | 1 | 0.00 | 0.40 | 0.22 | 0.17 |
| k__Bacteria\|p__Proteobacteria\|c__Gammaproteobacteria\|o__Enterobacteriales\|f__Enterobacteriaceae\|g__Enterobacter\|s__Enterobacter_cloacae | 0 | 2 | 0 | 2 | 0.00 | 0.40 | 0.00 | 0.33 |
| k__Bacteria\|p__Proteobacteria\|c__Gammaproteobacteria\|o__Enterobacteriales\|f__Enterobacteriaceae\|g__Klebsiella\|s__Klebsiella_pneumoniae | 0 | 3 | 1 | 5 | 0.00 | 0.60 | 0.11 | 0.83 |
| k__Viruses\|p__Viruses_noname\|c__Viruses_noname\|o__Viruses_noname\|f__Potyviridae\|g__Potyvirus\|s__Dasheen_mosaic_virus | 0 | 3 | 0 | 2 | 0.00 | 0.60 | 0.00 | 0.33 |

| **Supplementary** **Table 9.** Characteristics of the infants whose fecal samples were used in the fecal microbiota transfer experiments | | | | | | | | |
| --- | --- | --- | --- | --- | --- | --- | --- | --- |
| Neonatal | Sex | Gestational age (weeks) | Birthweight (grams) | Mode of delivery | Intrapartum antibiotics | Antibiotics before 6 months of age | Duration of breastfeeding (months) |  |
|  |  |  |  |  |  |  |  |  |
| antibiotics |  |  |  |  |  |  |  |  |
| 1 | M | 40^5/7^ | 4190 | VD | None | No | 8 |  |
| 2 | M | 38^1/7^ | 3640 | VD | penicillin G | No | 9 |  |
| 3 | F | 40^5/7^ | 3600 | VD | penicillin G | No | 5 |  |
| 4 | M | 36^0/7^ | 2950 | VD | None | No | 9 |  |
| 5 | M | 36^0/7^ | 2810 | VD | Penicillin G | Yes | 8 |  |
| Control |  |  |  |  |  |  |  |  |
| 1 | F | 41^4/7^ | 3930 | VD | None | No | 8 |  |
| 2 | M | 40^5/7^ | 3720 | VD | None | No | 9 |  |
| 3 | M | 41^3/7^ | 4370 | VD | None | No | 9 |  |
| 4 | M | 42^0/7^ | 4260 | VD | None | No | 8 |  |
| 5 | M | 41^1/7^ | 4460 | VD | None | No | 14 |  |
| 6 | M | 41^5/7^ | 3795 | VD | None | No | 8.5 |  |
| 7 | F | 39^2/7^ | 3870 | VD | None | Yes | 14 |  |
| 8 | M | 39^5/7^ | 3860 | VD | None | No | 12 |  |
| 9 | F | 39^5/7^ | 2930 | VD | None | No | 8 |  |
| 10 | F | 41^5/7^ | 3020 | VD | None | No | 12 |  |
| 11 | F | 37^4/7^ | 3640 | VD | None | No | 10 |  |
| *M=male, F=female, VD=vaginal delivery.* | | | |  |  |  |  | |
